# Response to ‘Eruptive keratoacanthomas associated with dupilumab therapy’

**DOI:** 10.1002/ski2.197

**Published:** 2023-03-02

**Authors:** Alexandra Patchinsky, Victoria Kalita, Philippe Muller, Nadine Petitpain

**Affiliations:** ^1^ Service de dermatologie CHR Metz‐Thionville Hôpital Bel Air Thionville France; ^2^ Service de pharmacovigilance CHRU de Nancy Nancy France

## Abstract

Case of a patient developing multiple rashes of squamous cell carcinoma 6 weeks after the introduction of dupilumab due to atopic eczema.

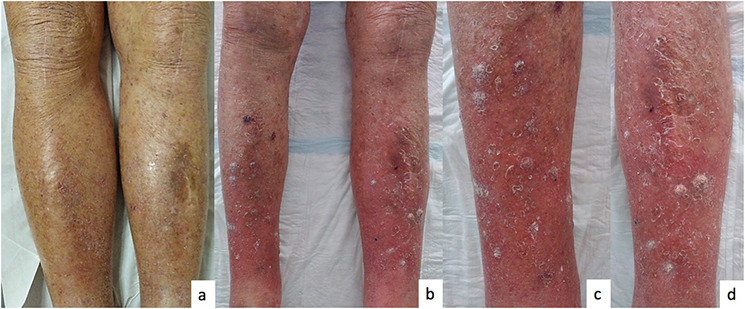

## ETHICS STATEMENT

Not applicable.

Dear Editor,

We read with interest the recent paper by Gleeson et al.^1^ presenting a case of eruptive keratoacanthomas associated with dupilumab therapy since we recently observed a similar case.

It concerns an 86‐year‐old patient with previous medical history of vulgaris ichthyosis, severe renal insufficiency, asthma and recent severe atopic dermatitis (clinical and biological diagnosis (total IgE 258 kU/L and hypereosinophilia 827 G/L)). Treatment with topical corticosteroids was insufficient and aggravated his severe dermatoporosis. Due to his severe renal insufficiency, neither methotrexate nor cyclosporin were prescribed for atopic dermatitis, but dupilumab therapy was started according to the usual scheme (initial subcutaneous injection with 600 mg followed by 300 mg on alternate weeks).

**FIGURE 1:**
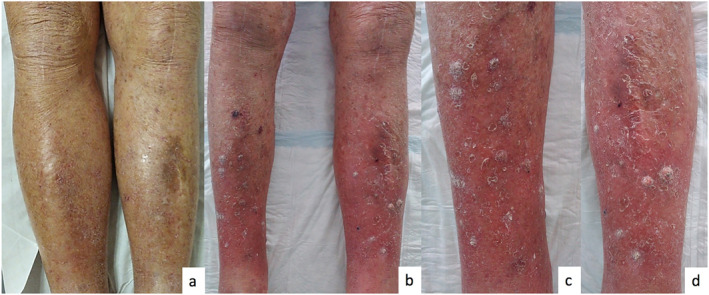
(a) Patient's legs before dupilmab; (b) patient's legs 2 months after dupilumab initiation; (c) right leg after dupilumab treatment; (d) left leg after dupilumab treatment

Within 6 weeks (four injections), the patient developed about 20 keratotic papules and some keratotic nodules over his legs and arms (Figure 1).

The nodules evoking infiltrating squamous cell carcinoma were surgically removed, whereas topical 5‐FU was applied for keratotic papules evoking non‐invasive squamous cell carcinoma. Histology of three nodules confirmed the diagnosis of invasive squamous cell carcinoma, all other lesions were clinically identical. It should be noted that the patient had no personal or family history of malignant skin lesions, intense photo‐exposure, previous ultraviolet therapy, immunosuppression or carcinoma‐inducing drugs in the past.

As no pruritic exanthema left, we stopped dupilumab therapy, however, new lesions continued to appear during the following month (12 weeks after the first dupilumab injection), requiring surgical or topical 5‐FU management. At the time of writing this letter, the patient is still under observation and new lesions may still develop.

Our query in the French pharmacovigilance database retrieved no similar case, but in Vigibase®, we identified 20 squamous cell carcinomas cases, four actinic keratosis cases and two keratocanthomas reported with dupilumab.

One possible pathophysiological mechanism is IL‐4, which is blocked by Dupilumab. IL‐4 induces the production of transforming growth factor (TGF)‐β, which acts as a cell cycle regulator by inhibition proliferation. Dupilumab therefore decreases TGF‐β production, which reduces the control of cell cycle proliferation, potentially leading to malignant proliferation.

In conclusion, we strongly support the conclusion of Gleeson et al. to monitor the onset of squamous cell carcinoma, actinic keratosis or keratocanthomas in dupilumab treated patient.

Further studies are necessary to investigate the role of dupilumab in the development of potentially malignant cutaneous lesions.

## CONFLICTS OF INTEREST

None to declare.

## AUTHOR CONTRIBUTIONS

**Alexandra Patchinsky**: Conceptualization (Equal); Writing – original draft (Equal). **Victoria Kalita**: Writing – review & editing (Equal). **Philippe Muller**: Validation (Equal). **Nadine Petitpain**: Investigation (Equal); Resources (Equal); Validation (Equal).

## FUNDING INFORMATION

This letter received no specific grant from any funding agency in the public, commercial, or not‐for‐profit sectors.

## Data Availability

Data sharing not applicable to this article as no datasets were generated or analyzed during the current study.
